# Where to Find Evidence-Based Information on Occupational Safety and Health?

**DOI:** 10.5334/aogh.3131

**Published:** 2021-01-06

**Authors:** Frank van Dijk, Yohama Caraballo-Arias

**Affiliations:** 1 Occupational Physician. Learning and Developing Occupational Health (LDOH) Foundation; emeritus professor in Occupational Health (The Netherlands). Leiden, Netherlands; 2 Occupational Physician. International Commission on Occupational Health (ICOH) Venezuelan National Secretary; Council of Scientific Advisors American College of Occupational & Environmental Medicine (ACOEM); Learning and Developing Occupational Health (LDOH) Foundation. Caracas, Venezuela

## Abstract

**Background:**

Occupational Safety and Health (OSH) professionals must base their advice and interventions on evidence from science, in balance with their expertise, and with workers’ and other stakeholders’ values and preferences. Evidence-based professional practice is one of the remedies against misinformation creating confusion and distrust in the society.

**Objectives:**

To present, for OSH professionals, an overview and critical considerations about concepts, strategies, and tools needed for an accurate search for evidence-based information.

**Methods:**

Information sources have been collected and discussed as a base for a documented vision on knowledge questions, online information sources, search engines, databases, and tools.

**Results:**

Every search should start with a carefully phrased question. To help finding a reliable answer, potential evidence-based online sources are presented. Systematic reviews and original scientific articles are regarded as primary sources. Secondary and tertiary sources are discussed, such as practice guidelines, point-of-care summaries, advisory reports, quality websites or apps, Wikipedia, quality videos, and e-lessons. To find sources, adequate use of search engines and databases is required. Examples are discussed briefly, such as PubMed/MEDLINE, Virtual Health Library, NICE, Cochrane Library, Cochrane Work, Google (Scholar), and YouTube.

**Conclusions:**

Evidence-based practice in OSH must be stimulated, relying mainly on trusted online sources. The breadth of appropriate information sources is wider than described in most publications. Search engines facilitate the finding of quality reports, videos, e-courses, and websites. Such sources can be explored by well-trained professionals to complement the use of scientific articles, reviews, point-of-care summaries, and guidelines. Adequate use of online information sources requires awareness, motivation, and skills in professionals and educators. To date, the quality of skills in searching is low, thus a more adequate education is crucial. The quality of sources, search engines, and databases will be considered more thoroughly in another study. International collaboration is profitable and needs new drivers.

## INTRODUCTION

Given the high demand in society for health and safety provisions at work based on reliable information, occupational safety and health (OSH) professionals should find their roots in evidence-based practice [1,2]. A new term is “evidence-informed decision making” [3]. A work definition of evidence-based practice could be: evidence-based practice is the use of evidence from literature, and other evidence-based sources, for advice and decisions that favor the health, safety, well-being, and work ability of workers. Therefore, evidence-based information must be integrated with professional expertise and the workers’ values [4]. Contextual factors must be considered related to legislation, culture, financial, and technical possibilities. Ethical considerations should be heeded.

Evidence-based performance is in favor of workers, their families, and the enterprises involved, and prevents unnecessary expenses for health care and social security.

The purpose of this article is to consider the basic concepts, strategies and tools needed for a faster and more accurate search for evidence-based information, thus saving time and money [5].

### PRINTED BOOKS OR ONLINE SOURCES

Books and manuals are useful for “background knowledge”, such as for the explanation of basic concepts and for comprehensive summaries of knowledge. Examples are the Practical Manual on the International Classification of Functioning, Disability and Health (ICF, WHO) [6], and the book “Agricultural Medicine: Rural Occupational and Environmental Health, Safety, and Prevention” [7].

**Figure 1:**
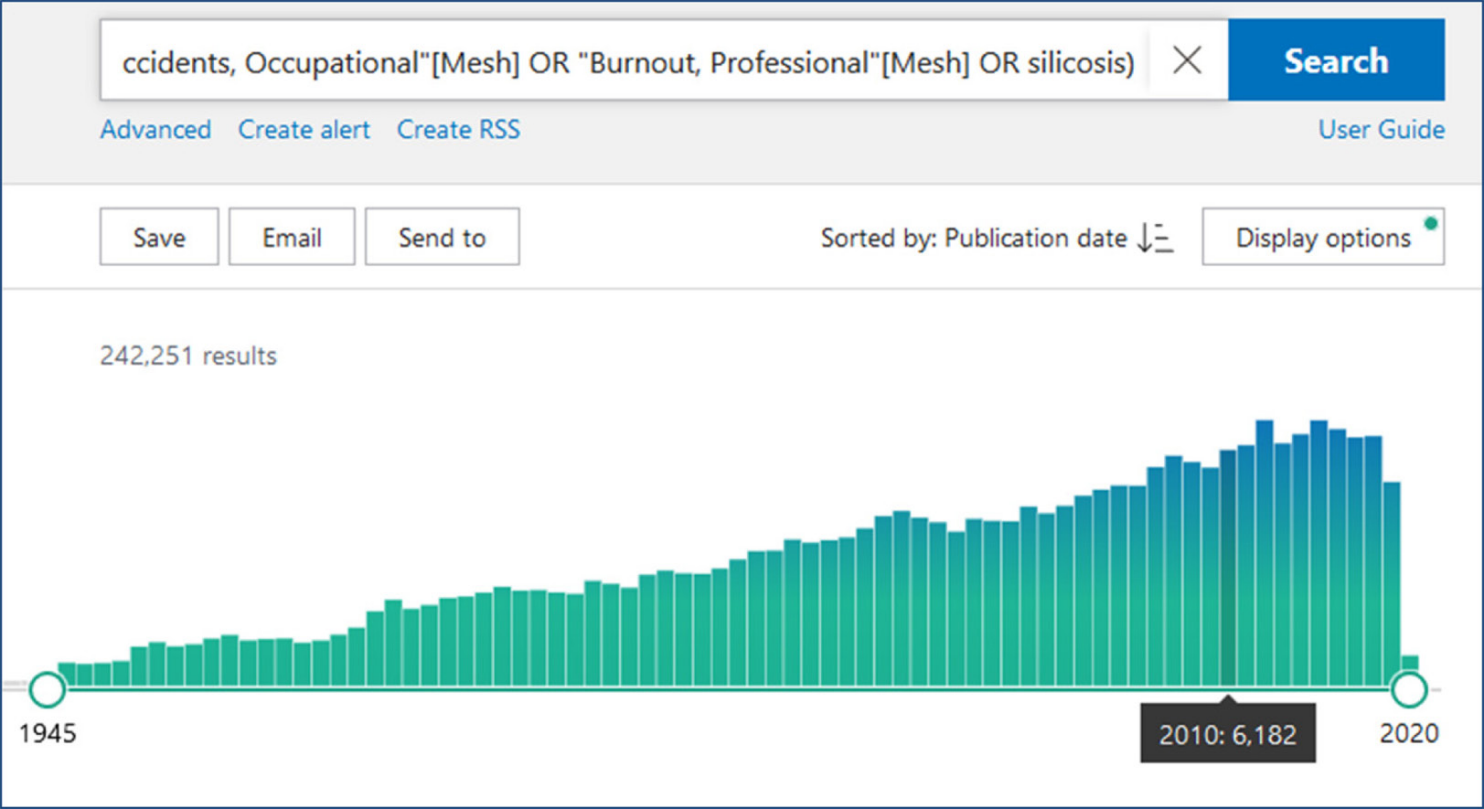
Number of articles on OSH topics in PubMed, per year, searching with a string of 13 OSH-related Medical Subject Headings (Mesh) terms [8].

Printed books, however, cannot cover all information from relevant quality articles, reviews, advisory reports, and guidelines. A PubMed search with a string of OSH-related MeSH (Medical Subject Headings) terms shows about 240,000 articles with a yearly increase of more than 6,000 articles (***Figure 1***) [8]. Regularly updated e-books, web-based point-of-care summaries, online guidelines, and reviews can better keep up with the newest scientific information than printed books can.

In practice, “foreground knowledge” is often needed, i.e. detailed knowledge focused on a specific worker or population, a specific disease, or intervention. Therefore, online accessible articles, reports, reviews, websites, or guidelines are often more appropriate in supporting decision making, than printed books. Up-to-date information is needed for advice and decisions in dynamic knowledge areas, such as for hazards in green jobs, health effects of nanoparticles, and effectiveness of e-health interventions.

### TRAINING OF OSH PROFESSIONALS IN EVIDENCE-BASED PRACTICE

Given an OSH issue, part of the information needed can be found through debriefing a worker or employer, or by collecting data at the workplace or elsewhere in the company. In other situations, professionals can look for available (online) information that goes beyond information directly taken from reality. We see that OSH professionals are formulating knowledge questions, when stimulated to improve the quality of their decision making [9]. A study suggests that evidence found in the literature is better than professional advice from a colleague, for instance [10]. Search skills, however, have to be trained.

Occupational physicians trained in literature searching on the etiology of occupational diseases performed better in the diagnosis than a control group [11]. Another example is the improvement of advice on sickness absence after literature searching, part of an EBM course embedded in case-method group learning [12,13]. Social insurance physicians also working in case-based EBM learning groups increased the percentage of evidence-based disability evaluations in practice [14]. E-learning can be effective as well. After participating in an e-learning module on occupational asthma, healthcare professionals reported increased knowledge, awareness, and use of evidence-based guidelines [15].

## A VARIETY OF QUESTIONS

For a focus, knowledge questions can be subdivided in categories. Classic medical examples are diagnostic, etiologic, intervention-related, prognostic, or descriptive/epidemiological questions (***Table 1***). In addition, other knowledge questions can be observed, such as on the quality of a specific instrument for risk assessment, on the effectiveness of various implementation strategies for a hearing conservation program, and on stakeholders’ opinions about ethical issues, such as genetic susceptibility testing to prevent occupational diseases.

**Table 1:** Categories of classic medical questions using COVID-19 pandemic as an example


Diagnostic questions:	Accuracy of rapid tests for COVID-19 in health care workers without symptoms

Etiologic questions:	Potential work-related exposure of receptionists facing (suspected) COVID-19 patients

Intervention questions (therapy, prevention, education)	Effectiveness of FFP2 face masks in physiotherapy to prevent COVID-19 infection

Prognostic questions	Prediction of the (long-term) course of disability in COVID-19 pneumonia, for elderly manual workers

Descriptive epidemiological questions	Prevalence of post-traumatic stress disorders in emergency workers treating COVID-19 patients


For intervention, etiologic, and prognostic questions, we promote the use of a **PICO (or PECO)** scheme to prioritize questions and to generate search terms. The **P** stands for the patient group or population, **I** for the intervention (or **E** for the exposure or prognostic factor), and **C** for the comparison. The **O** represents the outcome such as improved or deteriorated figures for a work-related disease, injury, disability, health, and well-being.

Example, the etiologic question is: Do sewage workers run a risk of getting an occupational disease?

**Table:** 

– **P**atient or population	“workers” or “sewage workers”
–**I**ntervention or **E**xposure	“sewage”
–**C**omparison	–––––
–**O**utcome	“occupational diseases”

The comparison term is occasionally used, for example, when comparing the effectiveness of a minimal versus a comprehensive intervention. In searches, we mostly use no more than two (groups of) search terms to avoid exclusion of relevant findings.

**Figure 2:**
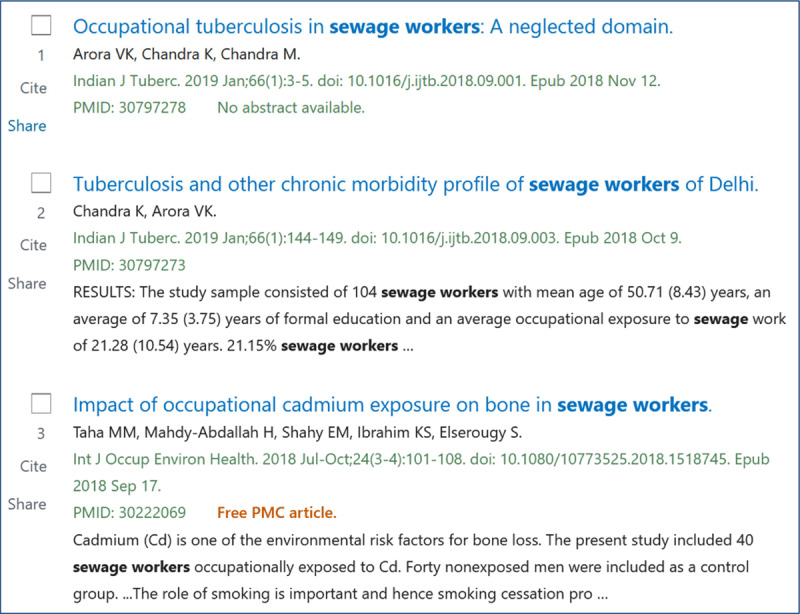
Screenshot of first three out of 48 titles in a PubMed search on occupational diseases in sewage workers (April 2020).

In a first inquiry we have chosen as search terms in PubMed: “sewage workers” AND “Occupational Diseases” [Mesh] (***Figure 2***). In a second inquiry we used the search terms “workers” AND “Sewage” [Mesh].

The findings in the first inquiry refer to diseases such as tuberculosis, helicobacter pylori infections, pulmonary diseases, effects of cadmium exposure, hepatitis A, B, and E. Likewise, the first inquiry found one review study, whereas, in the second inquiry, we found four other review studies.

In environmental health the **P** could represent communities living near a sewage plant. The **O** could be “environmental diseases” or “environmental exposure”. Search terms can be “sewage plant” and “Environmental Pollution” [Mesh]. In the search, studies are found on exposure to radioactivity via an effluent channel from iodine-131 patients in hospitals, on exposure to mercury, and to enteric viruses and bacteria [16].

## INFORMATION SOURCES

**Figure 3:**
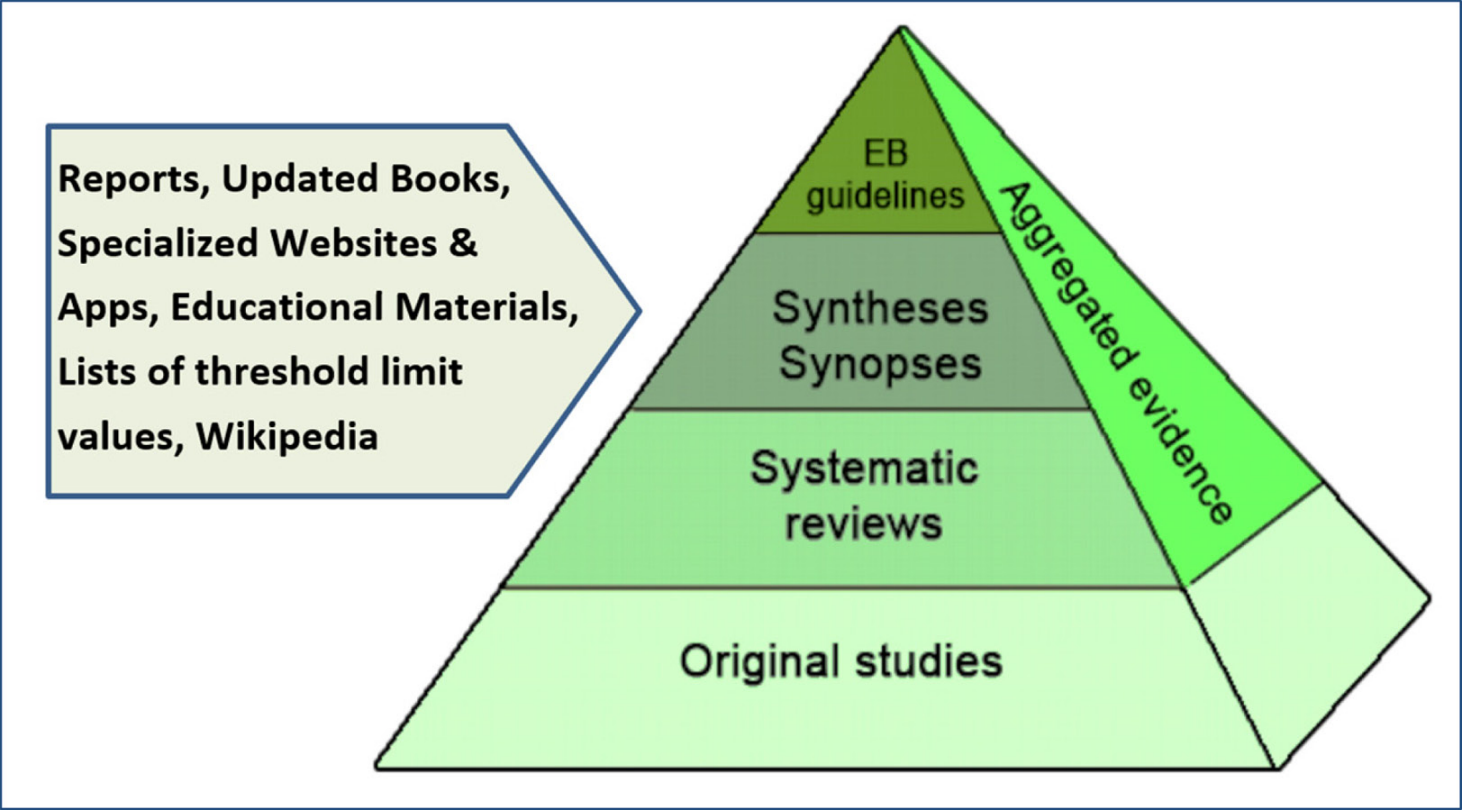
Search pyramid used in the Amsterdam University Medical Centre, adapted by the authors [17].

The search pyramid (***Figure 3***) shows different categories of sources and what to explore first [17]. The sources shown from top to bottom represent the ranking of usefulness for a professional in practice, not the order of level of evidence. Practitioners mostly start at the top, searching for evidence-based guidelines, and then move downwards. Problems might arise due to lack of open access to full texts [18].

### EVIDENCE-BASED PRACTICE GUIDELINES

Practice guidelines, when available, can be the first choice for practitioners, for example, for the writing of a protocol to prevent work-related dermatitis in a pharmaceutical production plant.

An interesting, yet a bit outdated guideline is “Dermatitis: Occupational aspects of management” (2009), a guideline from the UK [19].

Guidelines from the UK on occupational health issues can be found in National Health Service (NHS) Plus [20]. National Institute for Health and Care Excellence (NICE) presents medical guidelines potentially relevant for OSH [21]. A study concluded that occupational health professionals should participate more actively in developing NICE guidelines to integrate work-related issues [22]. In the Netherlands, such integration is carried out for many medical guidelines [23]. Guidelines on OSH issues developed in the USA can be accessed at the American College of Occupational and Environmental Medicine (ACOEM) website [24]. In Colombia, eight “Guidelines for comprehensive health care in evidence-based Occupational Health” (GATISO) were issued in 2007 [25]. Two Latin American guidelines on vaccinations for workers were recently published [26,27]. Dutch evidence-based guidelines on occupational health can be found online (29 guidelines on a wide variety of topics); a few are translated into English [28].

Guidelines for mental disorders and stress-related complaints are still a challenge [29,30]. There is no international database or e-library of OSH guidelines. Guidelines can be retrieved at PubMed adding “Practice Guideline [Publication Type]” to the search. Some guidelines require subscription fees.

### SYNTHESES, REPORTS, SPECIFIC WEBSITES/APPS, WIKIPEDIA

Syntheses are defined as critically appraised topics whereas synopses are critically appraised individual articles. The category of syntheses includes several summary-type sources.

Web-based point-of-care information summaries combine current evidence with expert guidance. Such syntheses are described as “web-based medical compendia designed to deliver predigested, rapidly accessible, comprehensive, periodically updated, and evidence-based information” [31]. To date, these summaries are mostly focused on hospital-based clinical health care. Under given conditions, they can offer direct access to a full text article. Users or institutions need to pay a subscription fee.

We explored UpToDate (www.uptodate.com) and Dynamed Plus (www.dynamed.com). UpToDate does not screen occupational health journals, so this tool is of limited value for our domain, aside from traditional not-work-related clinical questions. Dynamed Plus screens some common OSH journals but lacks an editorial board on occupational health.

The potential impact on OSH practice is high when OSH-related articles would be systematically included and when OSH expertise is involved. Good accessibility might be a problem because of a fee.

Reports can be of high quality when written by experts and can often be found using Google (add PDF in the search) or another search engine. An example is the “Information notices on occupational diseases: a guide to diagnosis” (EU, 2009) [32]. Another strategy is screening the site of an authoritative (international) institute, for example, International Agency for Research on Cancer (IARC). One can try sites of various leading (inter) national institutes and associations, such as Health and Safety Executive: HSE (UK); European Agency for Safety and Health at Work: EU-OSHA (EU), Fundacentro (Brazil); National Institute for Occupational Safety and Health: NIOSH (USA); Occupational Safety and Health Administration: OSHA (USA); Canadian Centre for Occupational Health and Safety: CCOHS (Canada); Safe Work Australia; World Health Organization (WHO); International Labour Organization (ILO); International Occupational Hygiene Association: IOHA; International Ergonomics Association: IEA; and International Commission on Occupational Health: ICOH (interdisciplinary).

About the carcinogenicity of diesel exhaust: typing “diesel cancer PDF” in Google gives, as first hits, on my PC in the Netherlands (Google personalized searches) the articles “Diesel engine emissions and lung cancer: insights from research design to policy” (Xu R et al., 2018) and “Diesel Exhaust Causes Lung Cancer – Now What?” (Silverman DT, 2017). Other findings include a press release of IARC (2012) classifying diesel engine exhaust as carcinogenic to humans and an expert advisory report, “Diesel Engine Exhaust” of the Health Council of the Netherlands (2019).

#### Websities and Apps

Websites can be visited directly using a list of quality sites (***Table 2***).

**Table 2:** Examples of quality websites for OSH.


Dermatology	DermNet (New Zealand)	*https://dermnetnz.org/*

Infectious diseases	GLEWS + (outbreaks)	*http://www.glews.net/*

Chemical substances	Hazmap	*https://haz-map.com*

	ATSDR [33]	*https://www.atsdr.cdc.gov/substances/index.asp*

Risk assessments	OIRA (EU)	*https://oiraproject.eu/es*

OSH questions	OSH Answers (Canada)	*https://www.ccohs.ca/oshanswers/*

**Education**		

OSH education in all schools	ENETOSH	*http://www.enetosh.net/*

Medical students	EMUTOM	*http://www.emutom.eu/*

OSH professionals	LDOH e-library	*https://library-education-osh.ldoh.net/*

Occup. Hygiene professionals	OHTA, IOHA	*http://www.ohlearning.com/*

OSH education, general	Geolibrary (Occup.)	*http://www.geolibrary.org/*


Only a few sites for professionals are adapted as apps, for example, the “NIOSH Mobile Pocket Guide to chemical hazards” and PubMed. A few instruments for professionals are available as apps, for example, the International Labour Organization (ILO) “Ergonomic Checkpoints”, “Ergonomic Checkpoints in Agriculture”, and “Stress Prevention at Work Checkpoints”. Websites for the general public are more often available as app, for example, “OSH answers” (CCOHS).

Websites and apps can be considered on the chosen target population, impacting the selected topics, complexity of the language, level of details, and use of references. Most sources in ***Table 2*** are aimed at professionals. In contrast, the Canadian CCOHS site including OSH Answers (FAQ), has been developed for the working population.

For education, the LDOH e-library focusses on OSH professionals and related students, whereas EMUTOM is exclusively produced for pre-graduate medical students. OHlearning has been created for students and professionals engaged in occupational hygiene, ENETOSH for developers of OSH education. Geolibrary covers a wide spectrum of users.

Wikipedia, offering about 5.5 million articles in English, is an important summarizing source of information for the global population, often linking directly to sources with detailed evidence-based information.

NIOSH (USA) actively collaborates with Wikimedia, contributing data and latest research so that the OSH information in Wikipedia is more complete, up-to-date, and free of errors. In return, Wikipedia brings many visitors to NIOSH and OSHA websites [34].

EU-OSHA developed OSHwiki, an online encyclopedia [35]. The OSHwiki community has created many articles written by “accredited authors” to ensure that the information is “current, reliable, and verifiable”. The page “COVID-19: Back to the workplace – Adapting workplaces and protecting workers” is available in 25 languages [36].

### REVIEWS AND SYSTEMATIC REVIEWS

**Figure 4:**
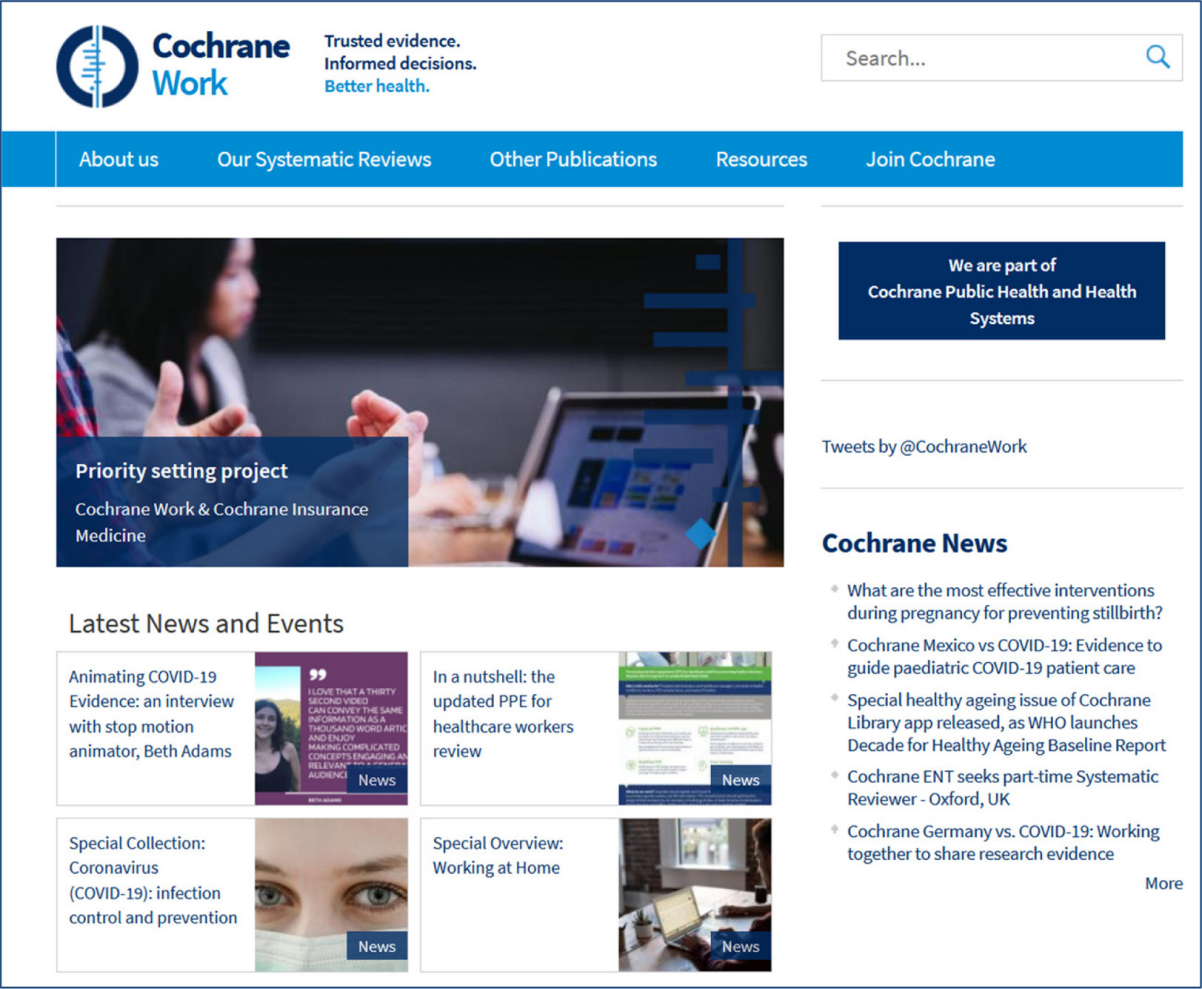
Cochrane Work website (*http://work.cochrane.org*).

The term “systematic” refers to a systematic approach described in extensive protocols dealing with formulating a question, collecting sources, distracting information, judging the quality, etc [37]. The Cochrane Library offers mainly access to systematic reviews on effectiveness of interventions. For OSH we recommend the Cochrane Work site (***Figure 4***). Virtual Health Library (VHL; BVS) and PubMed/MEDLINE also offer access to systematic reviews.

Scientific articles are often the primary (original) source of scientific information, mostly dealing with one specific topic within the broad interdisciplinary domain of OSH. Frequently, articles are the most relevant information source for a foreground question.

The number of scientific articles on OSH per year is ever growing (***Figure 1***). The topics reflect the actuality or popularity. For example, in the last five years, about 3,750 titles can be found in PubMed on professional burnout (“Burnout, Professional” [Mesh]), compared to roughly 400 titles on occupational dermatitis (“Dermatitis, Occupational” [Mesh]).

Let’s imagine an occupational physician, consulted by a nurse with dermatitis, assumes a contact allergy. A PubMed search (“Nurses” [Mesh] AND “Dermatitis, Allergic Contact” [Mesh]) gives 15 hits in the last 10 years. Various potential causes are mentioned: nickel, isopropanol-containing disinfectant, nitrile glove allergy caused by Pigment Blue 15, N-acetylcysteine, thiuram mix, and other chemical substances.

## SEARCH ENGINES AND DATABASES

The standard for scientific articles, reviews, and evidence-based guidelines is peer-review process that evaluates and improves its quality. Some databases only accept peer-reviewed sources. We present a few examples of search engines and databases (***Table 3***).

**Table 3:** Examples of resources (search engines and databases) relevant for OSH searches.


Free-of-charge resources **PubMed** accessing among others MEDLINE, database of biomedical scientific journals **Virtual Health Library (VHL, BVS)** accessing among others MEDLINE and databases of scientific journals in Spanish and Portuguese **Education Resources Information Center (ERIC)** a database of scientific journals on education **Google, Google Scholar** web search engines **YouTube** a search engine and database for videos Other search engines are available such as **Bing, Baidu, Yahoo! and Yandex**. Some newcomers may protect searchers’ privacy better such as **DuckDuckGo and Ecosia**

Resources that require a fee **Embase** (OVID) a biomedical database covering more drug and pharmacy journals than PubMed/MEDLINE **PsycInfo** a database of journals on behavioral sciences and mental health **CINAHL** a database of journals on nursing


We present some basic information on a few free-of-charge available resources.

PubMed (*http://www.ncbi.nlm.nih.gov/pubmed/*) provides access to biomedical literature, for example, present in MEDLINE containing about 30 million references. The search improves by using the “Medical Subject Headings” (MeSH terms), the “Advanced Search Builder”, and many “filters”. The tool “My NCBI” (National Center for Biotechnology Information) allows saving search strategies and selected titles and abstracts.

A study concluded that MEDLINE offered almost 90% of available quality intervention studies in occupational health [38]. Several OSH-specific search strings (filters) are available, supporting effective and efficient searching (***Table 4***).

**Table 4:** Search strings (filters) and strategies for MEDLINE on OSH topics.


For the prognosis of work ability	A search strategy to identify studies on the prognosis of work disability: A diagnostic test framework [39]

For agricultural workers’ diseases	PubMed search strings for the study of agricultural workers’ diseases [40].

For putative occupational determinants of a disease	Search strings for the study of putative occupational determinants of disease [41].

For return to work	Precision and recall of search strategies for identifying studies on return-to-work in Medline [42].

For the occupational origin of diseases	Developing search strategies in Medline on the occupational origin of diseases [43].

For chronic diseases and work participation	Searching bibliographic databases for literature on chronic disease and work participation [44].

For occupational health intervention studies	A search strategy for occupational health intervention studies [45].


Learning the search strategy on the etiology of suspected occupational diseases proved to be effective [11]. The occupational health intervention studies tool was effective in finding Italian language studies [46]. The Cochrane Work website offers a few other search strings and strategies.

The E-book “Occupational Safety and Health online. How to find reliable information” includes a do-it-yourself module to learn PubMed and a list of OSH terms for PubMed [5].

Virtual Health Library (VHL): Biblioteca Virtual en Salud, (BVS) [47] is a meta-search engine developed for Latin America that works in Portuguese, Spanish, and English. The library accesses 30 databases of information: LILACS, MEDLINE, Cochrane Library, WHOLIS and SciELO, among others.

All documents are assigned to the more than 30,000 DeCS terms (Descriptores en Ciencias de la Salud/Subject Descriptors in Health Sciences), equivalent to the MeSH terms of PubMed/MEDLINE. VHL (BVS) has a search tutorial and the option “Advanced Search”.

LILACS (Latin American and Caribbean Health Sciences Literature) [48] is a database of scientific literature on health covering almost 900 journals, monographs, and theses and containing 503,000 full texts (July 2020). The database uses the DeCS and offers an advanced search builder.

SciELO (Scientific Electronic Library Online) [49] is a model for the cooperative publication of scientific journals online (Open Access). SciELO promotes scientific communication in developing countries, particularly Latin America and the Caribbean. Free full text articles are available from more than 1,000 scientific journals from ten countries.

Google is a web search engine. The order of findings is the result of a popularity contest not associated with the evidence level. The engine incorporates academic and commercial sources and fake news. Users will likely get a huge number of hits and may feel seriously hindered by the amount of advertisements. Many hits are out of date and recent evidence-based articles, reviews, reports, and guidelines could be missed. Personalizing the search implies that results are unpredictable. In conclusion, a Google search can only be a complementary search, but Google is particularly strong in finding quality (inter) national reports (see Reports).

Try “Tools”: select “any time” and, for example, “past year”, to find recent information. Add the name of a reliable institute to the search. Add the abbreviations PDF or PPT to find reports, articles, and slides.

Google Scholar is a web search engine that catalogues millions of records of scholarly literature: academic and grey literature (articles, reports, theses, preprints, etc. not published by journal publishers using peer-reviewing). In an evaluation study, it was concluded that Google Scholar should not be used alone for systematic literature searches [50]. Another study concluded that using Google Scholar is time-consuming, shows only the first 1,000 hits, and entails a considerable risk of missing essential reliable publications [51]. Google Scholar cannot replace a professional search but can show a number of new sources.

Use “Sort by date” filtering only the past 12 months and the option “abstracts” to find recent academic articles [52].

YouTube is a search engine for videos showing academic e-lessons and technical instruction videos, but also one-sided commercial messages and fake news. Some selected videos may be interesting, especially for educational purposes.

Typing “silicosis construction” delivers attractive information on risks and control options in the construction industry. The Indian Association of Occupational Health distributed via YouTube 24 training videos for primary health care professionals on risks in various informal industries (type “IAOH – BOHS”).

## DISCUSSION AND CONCLUSIONS

The COVID-19 pandemic has shown how the parallel emerging “infodemic” can undermine global efforts to fight against the COVID-19 [53]. Good access to quality information by health care professionals is one of the remedies to this serious problem. Likewise, evidence-informed decision making should be the standard for OSH professionals in practice, in a balance with local conditions, workers’ values, and the professionals’ expertise.

The variety of appropriate online information sources is wider than often described. Search engines support the finding of quality reports, websites, e-lessons, and videos that can be used by well-trained professionals, complementary to scientific articles, reviews, syntheses, and guidelines.

Adequate use of online information sources by OSH professionals requires more awareness, skills, and sustained efforts of educators and professionals. Many professionals in OSH are only using Google to find information, not aware of the poor quality of their search techniques and findings. In general, they are hardly or not trained at all to find reliable information. OSH professionals and students must be educated thoroughly in finding and using online information effectively and efficiently. Several online courses are available [54,55]. We recommend a combination with face-to-face education within a blended approach [56].

The effectiveness of the searches presumes a combination of good search skills and high-quality resources. The quality of the sources, search engines, and databases may be considered in greater depth in a separate study.

A current issue for the OSH practice is the need to create practice guidelines, web-based point-of-care summaries, and systematic reviews dealing with the diversity of work-related and occupational diseases. The focus can be on the diagnosis, prognosis, therapy, and prevention, inclusive occupational health surveillance programs.

A good example that stimulates similar changes in OSH practice is the well-designed interventions in regional community health centers in Canada that have enabled these centers to work more evidence-informed [3]. International collaboration is fruitful as it has been demonstrated in the Cochrane Collaboration, the Guidelines International Network, Modernet [57] and many other international initiatives. Nevertheless, there is a need for new initiatives and for further financial resources leading to a more adequate knowledge infrastructure [58,59].

## AUTHOR CONTRIBUCTION

We confirm that both authors had access to the (online) sources and took part in writing and revising the manuscript.
